# Near-Infrared Quantum Cutting Long Persistent Luminescence

**DOI:** 10.1038/srep24884

**Published:** 2016-05-04

**Authors:** Zehua Zou, Lin Feng, Cheng Cao, Jiachi Zhang, Yuhua Wang

**Affiliations:** 1 Key Laboratory for Magnetism Magnetic Materials of the Ministry of Education, Lanzhou University, Lanzhou,730000, China

## Abstract

By combining the unique features of the quantum cutting luminescence and long persistent luminescence, we design a new concept called “near-infrared quantum cutting long persistent luminescence (NQPL)”, which makes it possible for us to obtain highly efficient (>100%) near-infrared long persistent luminescence in theory. Guided by the NQPL concept, we fabricate the first NQPL phosphor Ca_2_Ga_2_GeO_7_:Pr^3+^,Yb^3+^. It reveals that both the two-step energy transfer of model (I) and the one-step energy transfer of model (IV) occur in ^3^P_0_ levels of Pr^3+^. Although the actual efficiency is not sufficient for the practical application at this primitive stage, this discovery and the associated materials are still expected to have important implications for several fields such as crystalline Si solar cells and bio-medical imaging.

Quantum cutting luminescence (QCL) is an interesting and significant optical phenomenon occurring in materials that is able to convert one high-energy photon, generally at ultraviolet or visible wavelengths, into two low-energy photons, typically in near-infrared (NIR) spectral range^1^,^2^,^3^. As a schematic representation of the QCL illustrated in the left panel of Fig. 1, the donor ion (D) is excited to the emitting state (E) by absorbing one high-energy photon and then returns to the ground state (G) via an intermediate metastable state (M). The acceptor ion (A) is subsequently excited by the two-step energy transfer (ET) process, accompanied with two low-energy photons. In conventional QCL materials, many ions such as Pr^3+^ 2, Tb^3+^ 4, Tm^3+^ 5, Ho^3+^ 6, Er^3+^ 7, Nd^3+^ 8, Ce^3+^ 9, Eu^3+^ 10, Bi^3+^ 11 and Eu^2+^ 12 have been previously used as donor ions, owing to their ladderlike arranged energy levels that facilitate the photon absorption and subsequent ET steps. Yb^3+^ ion is generally used as acceptor ion due to its absorption and emission in NIR region, corresponding to ^2^F_5/2_ → ^2^F_7/2_ transition. Because the QCL process needs the low phonon frequency host to avoid the non-radiative losses, the present most efficient QCL has been achieved in some fluorides^2^,^4^,^5^.

Long persistent luminescence (LPL) is a phenomenon whereby the light emission can last for hours after the stoppage of the excitation sources^13^,^14^. A typical LPL process can be also qualitatively shown by a simple schematic diagram, as shown in the right panel of Fig. 1. Under light excitation, the ion is excited to a delocalized state (*D*; i.e., an excited state associated with delocalization properties). The delocalized electrons can be captured by and stored in traps for an appropriately long time^15^. After ceasing the excitation sources, the trapped electrons can escape back to the *D* state due to the thermal or photon stimulation, followed by the non-radiative relaxation to the *E* state and then radiative return to the ground *G* state, accompanied with LPL. Nowadays, the representative LPL materials include SrAl_2_O_4_:Eu^2+^,Dy^3+^ (green)^16^, CaAl_2_O_4_:Eu^2+^,Nd^3+^ (blue)^17^, Y_2_O_2_S:Eu^3+^,Mg^2+^,Ti^4+^ (red)^18^, and Zn_3_Ga_2_Ge_2_O_10_:Cr^3+^ (near-infrared: NIR)^19^.

Although the QCL and LPL processes show the different luminescence forms and mechanisms, they share similar implications for a variety of technologies as well. In particular, both QCL and LPL phosphors have attracted enormous attention in recent years for many applications, particularly as the down-converting materials to enhance efficiency of crystalline silicon (c-Si) solar cells and as the optical nanoprobes to increase sensitivity and depth of biomedical imaging^20^,^21^,^22^. However, both the QCL and LPL processes separately suffer from their own drawbacks. In QCL-based materials, the real-time excitation sources are always needed. In LPL-based materials, the down-converting efficiency is not sufficient for practical application.

The QCL and LPL diagrams in Fig. 1 naturally suggest that the drawbacks of the QCL and LPL processes in c-Si solar cell and biomedical imaging could possibly be overcome by combining the unique features of these two processes. Therefore, we propose a new conceptual luminescence process called “near-infrared quantum cutting long persistent luminescence (NQPL)” by combining two processes, as illustrated in the middle panel of Fig. 1. According to this new NQPL concept, one high-energy incident photon can promote the ion system to the delocalized state, followed by filling of the traps. When the stored energy is thermally released, two low-energy photons can be created via a quantum cutting channel (two-step ET). The net effect of the NQPL process is that the theoretical quantum efficiency of LPL may reach 200% in maximum, a very interesting phenomenon that has not been reported previously.

To justify the NQPL concept, we designed a new phosphor by codoping the acceptor ion (Yb^3+^) into an efficient LPL phosphor: Ca_2_Ga_2_GeO_7_:Pr^3+^, obtaining the first NQPL phosphor Ca_2_Ga_2_GeO_7_:Pr^3+^,Yb^3+^. The Ca_2_Ga_2_GeO_7_ host was selected because of the lower low phonon frequency (800–975 cm^−1^) of germanate, close to fluorides (500–600 cm^−1^)^23^,^24^.

## Results and Discussion

**Figure 2:**
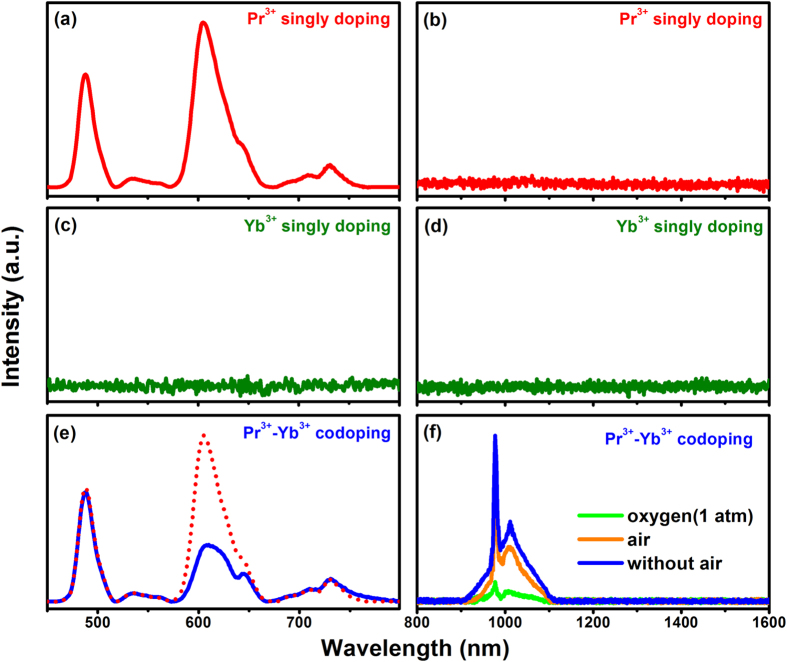
The LPL spectra of the Pr^3+^ (**a**,**b**), Yb^3+^ (**c**,**d**) single doped and Pr^3+^-Yb^3+^ codoped (**e**,**f**) samples recorded at 30 s after ultraviolet (UV) lamp (254 nm) irradiation for 15 min, the red dots are the LPL spectra of Pr^3+^ single doped sample for comparison and and the (**f**) shows the LPL spectra in different atmospheric preparation condition.

X-ray diffraction analyses show that the incorporation of the 0.1 mol% Pr^3+^ and 0.06–2 mol% Yb^3+^ ions into the Ca_2_Ga_2_GeO_7_ host does not induce obvious impurity, but the some impurity peaks arise when the content of Yb^3+^ is more than 2 mol% (see Fig. S1 in the supplemental material). Figure 2 shows the LPL spectra of the Pr^3+^ (a-b), Yb^3+^ (c-d) single doped and Pr^3+^-Yb^3+^ codoped (e-f) samples recorded after ultraviolet (254 nm) lamp irradiation for 10 min and again after a delay of 60 s. The LPL emissions due to ^3^P_0_ and ^1^D_2_ levels of Pr^3+^ in Ca_2_Ga_2_GeO_7_:Pr^3+^ sample can be clearly observed. The occurrence of ^3^P_0_ emissions of Pr^3+^ is very significant for the QCL of the Pr^3+^-Yb^3+^ pairs, and it should be associated with the low phonon energy of the Ca_2_Ga_2_GeO_7_ crystal, which partly prevents the multiphonon relaxation from ^3^P_0_ to ^1^D_2_. On the contrary, the NIR LPL of Yb^3+^ in the Ca_2_Ga_2_GeO_7_:Yb^3+^ sample is not recorded. Therefore, it can be clearly concluded that the characteristic NIR LPL (977 nm) of Yb^3+^ in the Ca_2_Ga_2_GeO_7_:Pr^3+^,Yb^3+^ sample which can last for more than 100 h must originate from the ET of Pr^3+^ → Yb^3+^. As shown in Fig. 2(e), the LPL band in range 560–660 nm due to ^1^D_2_ → ^3^H_4_, ^3^P_0_ → ^3^H_6_ and ^3^P_0_ → ^3^F_2_ transitions clearly reduces in intensity after codoping Yb^3+^, and it further suggests that the ET of Pr^3+^ → Yb^3+^ mainly originates from ^3^P_0_ and ^1^D_2_ levels of Pr^3+^. LPL in rare-earth ion doped crystals and glasses is a complex process and might be dependent on a combination of host-dopant defect state energy exchange. Thus, the samples were prepared in different atmospheric conditions including oxygen (1 atm), air and without air to gain the information of defect shown in Fig. 2(f)^25^,^26^. The intensity of LPL decreases with the partial pressure of oxygen increases. It is obvious that the LPL derives from the defect of vacancy oxygen, and the higher concentration of vacancy oxygen, the more traps in the bandgap which strengthen the intensity of LPL.

**Figure 3:**
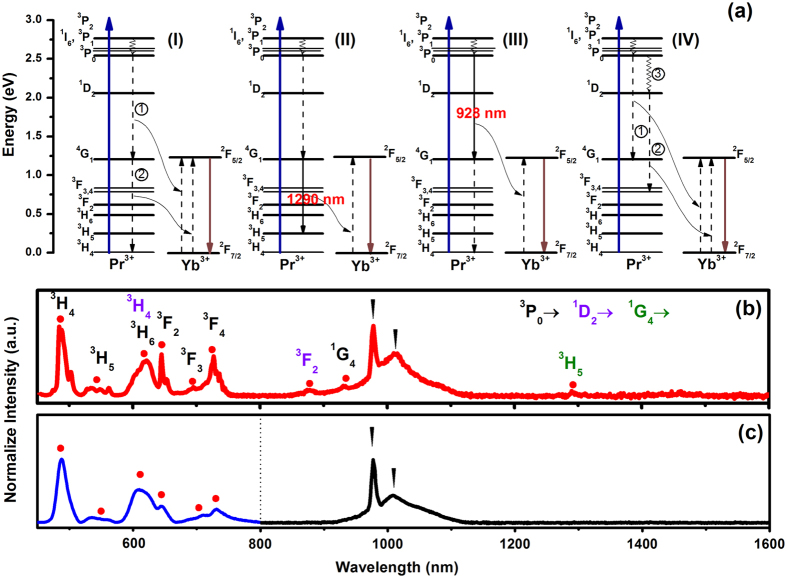
Four possible models of energy transfer mechanisms for the Pr^3+^ → Yb^3+^ pairs (**a**), photoluminescence spectra (**b**) and LPL (**c**) spectra of Ca_2_Ga_2_GeO_7_:Pr^3+^,Yb^3+^ by UV lamp irradiation.

Generally, four possible models of ET mechanisms for the Pr^3+^ → Yb^3+^ pairs should be taken into consideration as shown in Fig. 3: Model (I) two-step ET from Pr^3+^ to Yb^3+^ as Pr^3+^ (^3^P_0_ → ^1^G_4_)  →  Yb^3+^ (^2^F_5/2_ → ^2^F_7/2_) (①) and Pr^3+^ (^1^G_4_ → ^3^H_4_) → Yb^3+^ (^2^F_5/2_ → ^2^F_7/2_) (②), which results in the generation of two NIR photons by absorbing one photon; Model (II) and (III) refers to one-step ET from Pr^3+^ to Yb^3+^, which results in generation of two photons via Pr^3+^ (^3^P_0_ → ^1^G_4_) → Yb^3+^ (^2^F_5/2_ → ^2^F_7/2_), accompanied with Pr^3+^ (^1^G_4_ → ^3^H_5_) or Pr^3+^ (^1^G_4_ → ^3^H_5_)  →  Yb^3+^ (^2^F_5/2_ → ^2^F_7/2_), accompanied with Pr^3+^ (^3^P_0_ → ^1^G_4_); Model (IV) is one-step ET from Pr^3+^ to Yb^3+^ as Pr^3+^ (^3^P_0_ → ^1^G_4_) → Yb^3+^ (^2^F_5/2_ → ^2^F_7/2_) (①) and one-step ET of Pr^3+^ (^1^D_2_ → ^3^G_3,4_) → Yb^3+^ (^2^F_5/2_ → ^2^F_7/2_) (②) after multiphonon relaxation (^3^P_0_ → ^1^D_2_) (③). To make clear understanding of the ET mechanisms in this case, a wide range spectral investigation including NIR region is indispensable. Figure 3(b,c) depict the photoluminescence (PL) and LPL spectra of the optimal Ca_2_Ga_2_GeO_7_:Pr^3+^,Yb^3+^ sample. It can be seen that the PL (λ_ex_ = 254 nm) and LPL (after 254 nm irradiation) spectra are highly similar to each other, and the only difference is the observation of weak emission peaks at 928 nm (Pr^3+^:^3^P_0_ → ^1^G_4_) and 1290 nm (Pr^3+^:^1^G_4_ → ^3^H_5_), corresponding to model (II) and (III) in PL spectrum. However, because these emission peaks are very weak in PL spectrum and are completely not observed in LPL spectrum as shown in Fig. 3(b,c), the occurrence of the model (II) and (III) can be actually ignored in this discussion. Significantly, it is found that both the PL and LPL spectra consist of the characteristic emission from ^1^D_2_ level: 600 nm (^1^D_2_ → ^3^H_4_), indicating the existence of multiphonon relaxation form ^3^P_0_ to ^1^D_2_, i.e., model (IV). Because the NIR emission (977 nm) is the only characteristic of model (I), it can not be excluded or included at this stage. According to the mechanisms of models (I) and (IV), it is known that the ET to Yb^3+^ is efficient from both the ^3^P_0_ and the ^1^D_2_ levels of Pr^3+^. From the ^3^P_0_ level, a two-step ET or multiphonon relaxation may occur, while from the ^1^D_2_ level, resonant ET to Yb^3+^ is possible through a one-step ET process: Pr^3+^ (^1^D_2_ → ^3^F_3,4_) → Yb^3+^ (^2^F_5/2_ → ^2^F_7/2_). Note that in the proposed mechanisms, absorption of one photon to the ^3^P_*J*_ or higher ^1^I_6_ levels may be followed by the emission of two photons (977 nm); absorption to ^1^D_2_ would result in emission of only one 977 nm photon.

**Figure 4:**
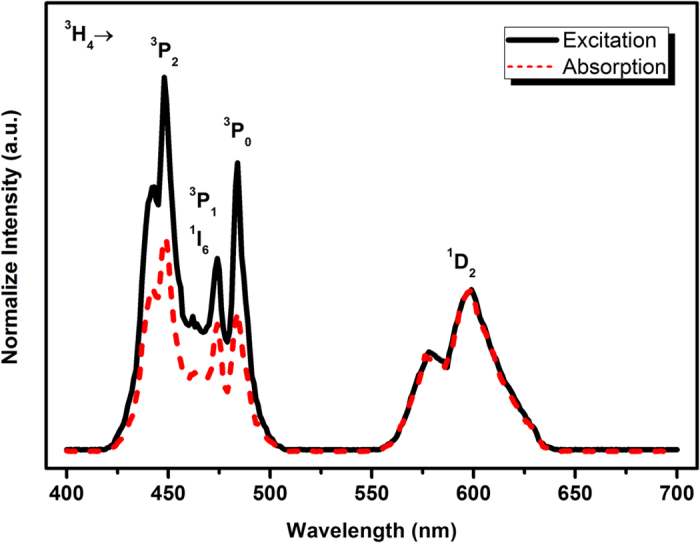
Excitation spectra monitored at 977 nm (black line) and absorption spectra (red line) for for Ca_2_Ga_2_GeO_7_:Pr^3+^,Yb^3+^.

By comparing the relative absorption strengths of the ^3^H_4_ → ^3^P_*J*_, ^3^H_4_ → ^1^I_6_, and ^3^H_4_ → ^1^D_2_ transitions with the corresponding relative photon fluxes in the excitation spectrum, the occurrence of the possible quantum cutting effect i.e., model (I) can be determined^2^. In Fig. 4, the normalized excitation (black line) and the diffuse reflectance (red line) spectra are shown for the Ca_2_Ga_2_GeO_7_:Pr^3+^,Yb^3+^ sample. The excitation spectrum is monitored by Yb^3+^ emission (977 nm). It can be seen that the area ratio (R_E_) of the ^3^P_J_ band to the ^1^D_2_ band in the excitation spectrum is 1.90, while that (R_A_) of the absorption spectrum is 1.15. When we assume that the quantum efficiencies to Yb^3+^ from ^3^P_J_ and that from ^1^D_2_ (100%) are equivalent, the emission intensity of Yb^3+^ ions by excited ^3^P_J_ levels should be also 1.15 times as strong as that of the ^1^D_2_ level. In fact, it is found in Fig. 4 that the excitation intensity by ^3^P_*J*_ is 1.90 times greater than that by ^1^D_2_, and this is direct evidence of quantum cutting as indicated in the model (I) of Fig. 3(a)^2^. However, if the model (I) is the only channel, the ratio of R_E_/R_A_ should be 2 in theory. The roughly estimated value of 1.65 indicates that both the two-step ET of model (I) and the one-step ET of model (IV) occur in the Pr^3+^-Yb^3+^ codoped samples. As mentioned earlier, both the PL and LPL occurs through the direct recombination of the conduction electrons with the emission centers, and the only difference is that the electrons in the conduction band originate from direct excitation in PL or from traps in LPL. Both the processes are achieved through conduction band, and the electrons would finally reach the ^3^P_0_ level of Pr^3+^ via relaxation. Therefore, the electrons in the ^3^P_0_ level face the same choice in PL and LPL processes (also evidenced by the highly similar spectra profiles). At this stage, the occurrence of the QCL and NQPL at ^3^P_0_ levels of Pr^3+^ can be demonstrated. Note that the actual quantum efficiency should be lower than the theoretical value of 165% due to the quenching effect, which reduces the Yb^3+^ emission. An estimate of the overall ET efficiency, which is the fraction of ^3^P_0_ excited states that relax through ET rather than radiative decay, can be obtained from the integrals under the normalized fluorescent decay curves, as outlined in refe. 27. From the fluorescent decay curves in Fig. S2, it is determined that the roughly estimated ET efficiency from ^3^P_0_ level including the one-step and the two-step processes is only 17.7% for the optimal Ca_2_Ga_2_GeO_7_:Pr^3+^,Yb^3+^ sample, and thus the actual quantum efficiency should be less than 117.7%. The low ET efficiency may be due to the low quenching concentration of Yb^3+^ in this host. As previously mentioned, when the codoping content of Yb^3+^ is more than 2 mol%, some impurities clearly arise and thus badly quench the NIR emission of Yb^3+^ (Fig. S1). However, although the efficiency is not sufficient for the practical applications at this primitive stage, this study is of significance both in the theoretical research on NQPL and in the future developmental practices of the crystalline Si solar cells and the biomedical imaging.

**Figure 5:**
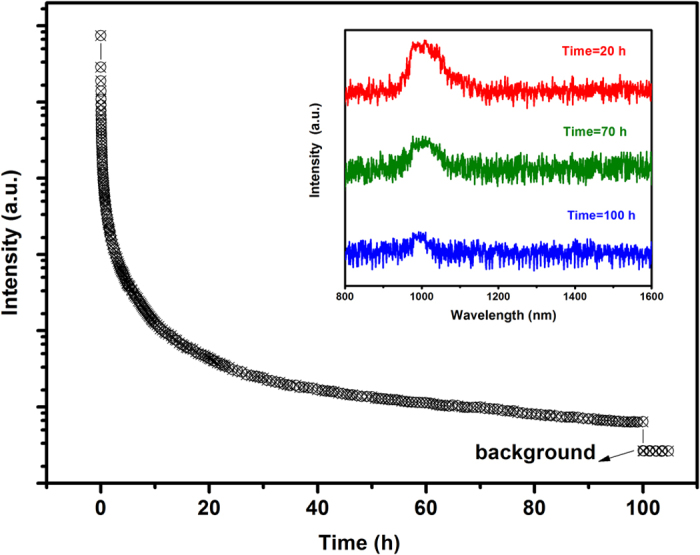
Near infrared (NIR) LPL decay curves of Ca_2_Ga_2_GeO_7_:Pr^3+^,Yb^3+^ monitored at 977 nm by UV lamp irradiation for 15 min and the NIR LPL spectra for different decay time (inset).

Additionally, the LPL duration time is also significant for the applications in the c-Si solar cells and biomedical imaging, and thus it is necessary to measure the LPL time of this material. Generally speaking, the duration time of visible LPL could be evaluated by the 0.32 mcd/m^2^, a value commonly used by the safety signage industry (about 100 times the sensitivity of the dark-adapted eye)^28^. However, NIR LPL is less efficiently sensed by the human eye. Instead, radiance is more appropriate than luminance for the evaluation of NIR LPL^29^. According to previous practices^19^,^30^, the NIR LPL around 977 nm of this materials could be recorded for more than 100 hours after irradiated for 15 min as shown in Fig. 5, although after such time from the end of the irradiation, the signal-to-noise ratio was strongly reduced making the Yb^3+^ emission barely detectable. The inset of Fig. 5 also gives the LPL spectrum acquired at different decay time. It is reasonable that the detectability of the NIR LPL at a given time strongly depends on the experimental conditions.

**Figure 6:**
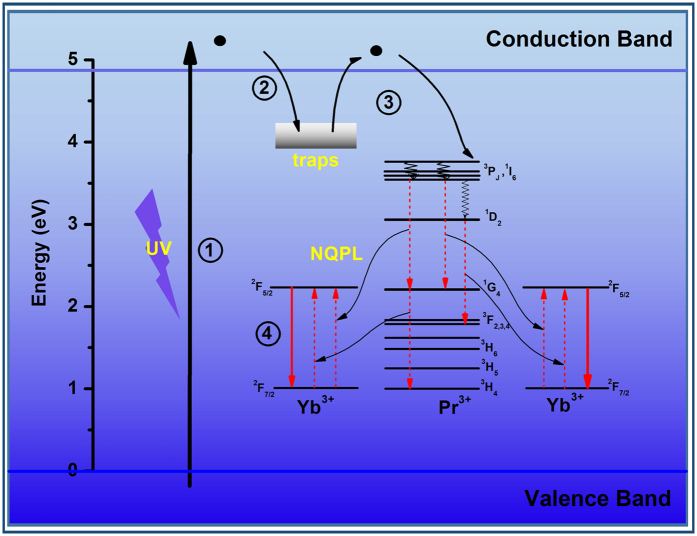
Schematic diagram of the NIR LPL mechanism for Ca_2_Ga_2_GeO_7_:Pr^3+^,Yb^3+^.

Accordingly, Fig. 6 exhibits a schematic representation of the NQPL mechanism. The trap levels continuously distribute over a wide range of energies and localize near the Pr^3+^ sites. Under ultraviolet light excitation, the electrons can be promoted to the conduction band (process ①). The electrons are subsequently captured by the traps below conduction band (process ②). The captured electrons are gradually released from the traps and are backtracked to the excited ^3^P_0_ level of Pr^3+^ via the conduction band (process ③). Finally, the energy is transferred from Pr^3+^ to Yb^3+^ via the one-step (model IV) and the two-step (model I) ET processes, and gives the NIR LPL of Yb^3+^ (④).

In summary, A new NQPL concept by combining the unique QCL and LPL processes is proposed for the first time. According to this idea, we designed the first NQPL phosphor Ca_2_Ga_2_GeO_7_:Pr^3+^,Yb^3+^ by incorporating acceptor Yb^3+^ ions into the LPL phosphor Ca_2_Ga_2_GeO_7_:Pr^3+^. It reveals that a two-step ET process from Pr^3+^ (^3^P_0_ → ^1^G_4_) → Yb^3+^ (^2^F_5/2_ → ^2^F_7/2_) and Pr^3+^ (^1^G_4_ → ^3^H_4_) → Yb^3+^ (^2^F_5/2_ → ^2^F_7/2_) occur in this phosphor, demonstrating the occurrence of the QCL and NQPL in ^3^P_0_ levels of Pr^3+^. Even though the actual QC efficiency still need to be improved, this interesting discovery enables the Ca_2_Ga_2_GeO_7_:Pr^3+^,Yb^3+^ phosphor to find potential applications in many important areas, particularly in c-Si solar cells and biomedical imaging that requires highly efficient, less environmental limitation, super-long and near-infrared LPL.

## Methods

### Synthesis

All phosphors were fabricated by a simple solid-state method. Stoichiometric amounts of CaCO_3_ (A.R.), Ga_2_O_3_ (A.R.), GeO_2_ (A.R.), Pr_6_O_11_ (4N) and Yb_2_O_3_ (4N) were used as starting materials. The ingredients were ground homogeneously in an agate mortar with anhydrous alcohol. Then the mixtures were sintered at 1573 K for 2 h in air (or oxygen (1 atm) and without air). After cooled down to room temperature, the final products were obtained.

### Characterization

The X-ray diffraction patterns were obtained on a Rigaku D/max-2400 powder diffractometer by using Cu Kα radiation at 40 kV and 60 mA. The luminescence decay curves were measured by a FLS-920T fluorescence spectrophotometer with a nF900 microsecond flashlamp as the light source. The photoluminescence and the long persistent luminescence spectra were recorded by FLS-920 fluorescence spectrophotometer (Edinburgh Instruments). The absorption spectra were recorded by a PerkinElmer Lambda 950 spectrometer in the region of 400–700 nm, while BaSO_4_ was used as a reference.

## Additional Information

**How to cite this article**: Zou, Z. *et al.* Near-Infrared Quantum Cutting Long Persistent Luminescence. *Sci. Rep.* **6**, 24884; doi: 10.1038/srep24884 (2016).

## Supplementary Material

Supplementary Information

## Figures and Tables

**Figure 1:**
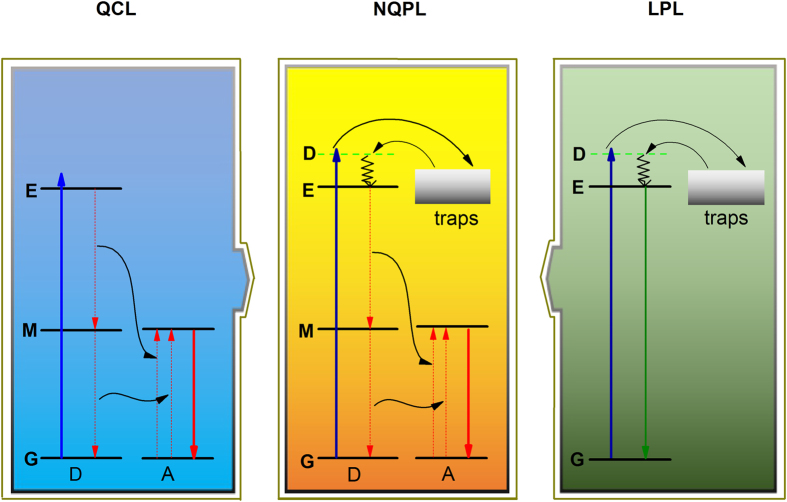
Schematic diagram of quantum cutting luminescence (QCL), near-infrared quantum cutting long persistent luminescence (NQPL), and long persistent luminescence (LPL).
