# Supplementary material for: Near-Infrared Quantum Cutting Long Persistent Luminescence
Source: Sci Rep. 2016 May 4;6:24884. doi: 10.1038/srep24884 (PMC4855154; doi:10.1038/srep24884)
Supplement: Supplementary Information [file srep24884-s1.doc]

**Near-Infrared Quantum Cutting Long Persistent Luminescence**

**Zehua Zou ^1^, Lin Feng ^1^, Cheng Cao ^1^, Jiachi Zhang ^1,*^ , Yuhua Wang ^1^**

^1^ Key Laboratory for Magnetism Magnetic Materials of the Ministry of Education, Lanzhou University, Lanzhou,730000, China

^*^ corresponding author: [zhangjch@lzu.edu.cn](mailto:zhangjch@lzu.edu.cn)

**Supplementary Information**


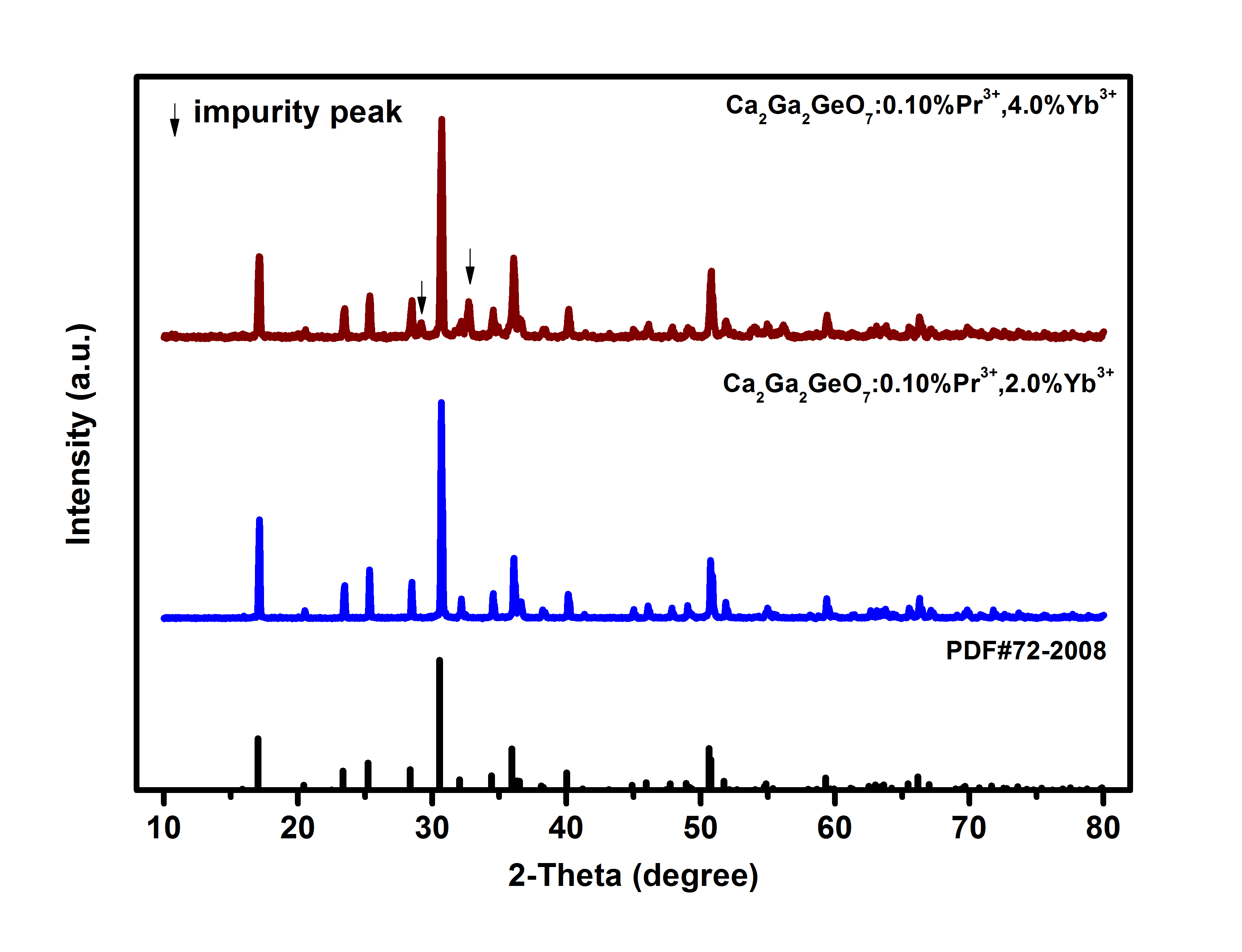


**Figure S1.** X-ray diffraction patterns of different concentration of Yb^3+^ ions prepared at 1300 ℃ for 2 h, and the JCPDS card.


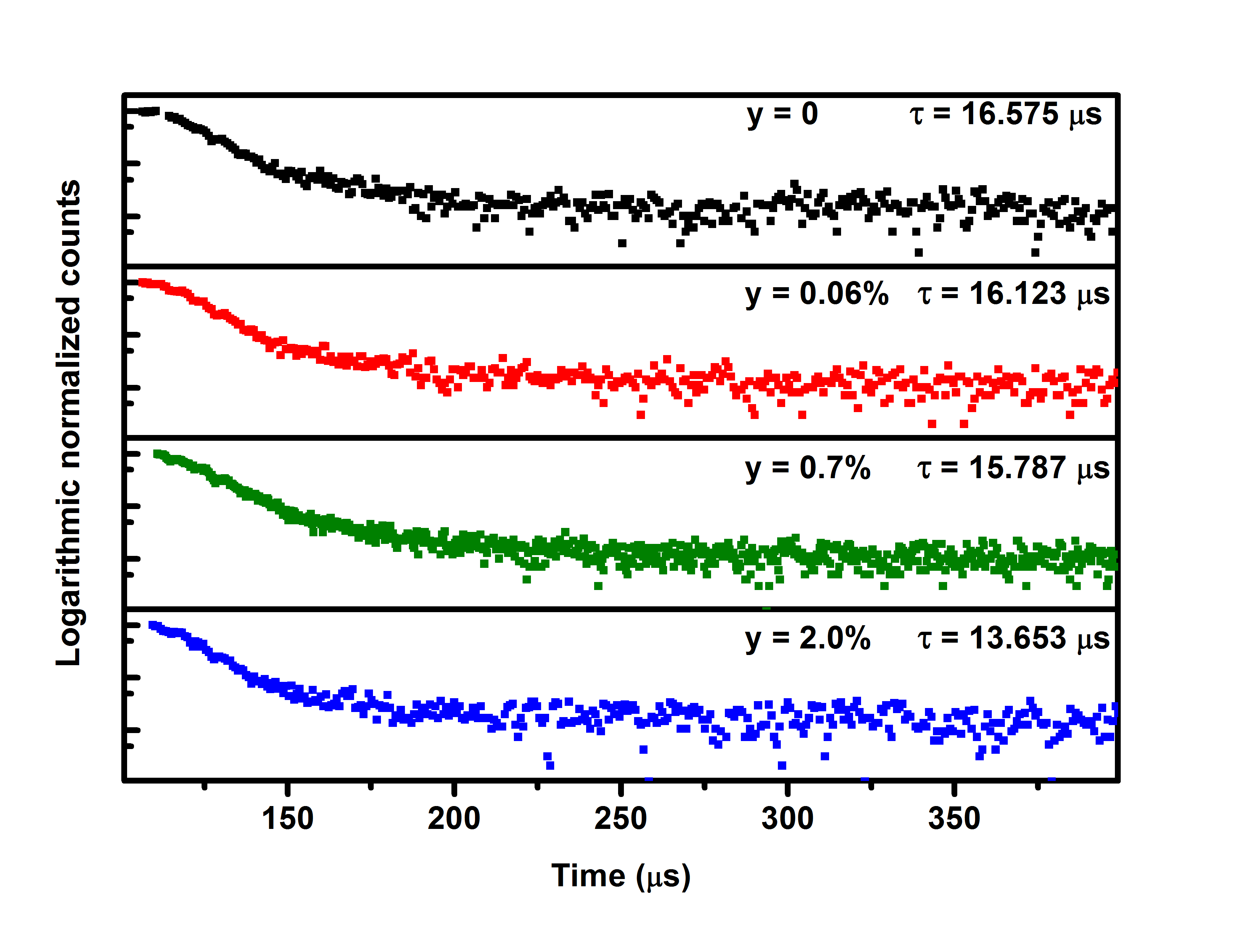


**Figure S2.** Luminescence decay curves of the Pr^3+^: ^3^P_0_ emission (^3^P_0_→^3^H_4_) in Ca_2_Ga_2_GeO_7_:Pr^3+^,yYb^3+^ (y=0, 0.06%, 0.7%, 2.0%) by 449 nm excitation.
